# The effects of eccentric cycling on vascular reactivity

**DOI:** 10.3389/fphys.2025.1554054

**Published:** 2025-03-19

**Authors:** Manuel Gomez, Samuel Montalvo, Daniel Conde, Gabriel Ibarra-Mejia, Alvaro N. Gurovich

**Affiliations:** ^1^ Clinical Applied Physiology Laboratory, Department of Physical Therapy and Movement Science, The University of Texas at El Paso, El Paso, TX, United States; ^2^ BioErgonomics Laboratory, Department of Public Health Sciences, The University of Texas at El Paso, El Paso, TX, United States; ^3^ Stanford Sports Cardiology, Division of Cardiovascular Medicine, Stanford University School of Medicine, Palo Alto, CA, United States; ^4^ Wu Tsai Human Performance Alliance, Stanford University, Palo Alto, CA, United States

**Keywords:** eccentric cycling, endothelial function, flow-mediated dilation, exercise-induced, endothelial shear stress

## Abstract

**Purpose:**

Eccentric cycling has gained attention as a novel exercise modality that increases muscle performance at lower metabolic demand, which could enhance cardiovascular rehabilitation. However, endothelial function response to eccentric cycling (ECC) has yielded contradictory results. Therefore, the purpose of this study was to determine the effect of 30 min of moderate-intensity cycling ECC on endothelial function.

**Methods:**

A total of 15 (9 females, 6 males) young, apparently healthy participants were recruited for two laboratory visits. First, a maximum oxygen consumption (VO_2_max) and blood lactate (BLa) threshold were measured to determine moderate workload intensity, followed by a familiarization stage on an ECC ergometer. During the second visit, a 30-min of moderate ECC was performed 72 h after the first visit. Endothelial function was measured via Flow-Mediated Dilation (FMD) pre- and post-exercise bout. FMD was calculated following traditional recommendations and adjusting for exercise-induced endothelial shear stress (ESS), utilizing the same pre-exercise baseline artery diameter for post-exercise FMD calculations.

**Results:**

There was a significant increase in endothelial function (p = 0.037) when adjusting pre-exercise baseline diameter to adjust for ESS, but when utilizing the traditional method no change in endothelial function was observed.

**Conclusion:**

30-min of moderate ECC showed a significant improvement in endothelial function when accounting for exercise-induced ESS. These results support the use of the pre-exercise baseline diameter when calculating post-exercise FMD to avoid the exercise-induced ESS or muscle metabolites effects on post-exercise artery diameter.

## Introduction

Cardiovascular disease (CVD) remains the leading cause of mortality worldwide, responsible for approximately 19 million deaths in 2020 (Tsao et al., 2022). Atherosclerosis, responsible for 90% of CVD cases globally, is primarily driven by endothelial dysfunction, characterized by a decrease in nitric oxide (NO) bioavailability (Allen et al., 2006; Flammer et al., 2012; Ghisi et al., 2010; Rosamond et al., 2007). This dysfunction is a critical factor in the development and progression of CVDs. When the endothelium, the inner lining of blood vessels, becomes dysfunctional, it can lead to impaired vasodilation, increased inflammation, and the formation of atherosclerotic plaques, which can restrict blood flow up to 50% (Allen et al., 2006; Ghisi et al., 2010). These plaques increase the risk of heart attacks, strokes, and other CV events.

Endothelial function can be assessed non-invasively via Flow-Mediated Dilation (FMD), which is a strong predictor of CV events in patients with established CVD (Thijssen et al., 2011) and measures endothelium-derived NO bioavailability (Harris et al., 2008; Thijssen et al., 2011). Even though FMD is considered a bioassay for endothelial function, discrepancies in FMD response have been noted following acute exercise with studies reporting both reductions and no changes, suggesting a time-dependent, intensity, and modality effects (Cosio-Lima et al., 2006; Goel et al., 2007; Harris et al., 2008; Johnson et al., 2012; Thijssen et al., 2006; Tinken et al., 2009a; Zhu et al., 2010). However, many of these studies have not considered the effect of exercise-induced endothelial shear stress (ESS) on post-exercise baseline vessel diameter or other normalizing techniques such as allometric scaling, which could influence the FMD calculations and, therefore, FMD interpretation.

Eccentric cycling (ECC) has emerged as a possible exercise alternative to treat chronic conditions such as heart failure (Chasland et al., 2017) or chronic obstructive pulmonary disease (Inostroza et al., 2022). ECC’s metabolic demands are lower than those of traditional aerobic exercise while improving health and fitness (Abbott et al., 1952; Dufour et al., 2007; Dufour et al., 2004; González-Bartholin et al., 2019; Knuttgen et al., 1982; Peñailillo et al., 2014; Peñailillo et al., 2013; Peñailillo et al., 2015; 2017; Peñailillo et al., 2018; Peñailillo et al., 2022; Perrey et al., 2001). However, the effect of ECC on endothelial function remains unclear. For example, Haynes et al. reported conflicting results in endothelial function after an acute bout of ECC exercise at matched external workloads (∼70% of maximum Watts), with variations attributed to artery size adjustments (Haynes et al., 2017). In addition, Stacy et al. and Rakobowchuk et al. observed a decrease in FMD (%) after 1 hour of strenuous eccentric resistance exercise and ECC below the first ventilatory threshold (∼33% of the maximal oxygen uptake) eccentric exercise (Caldwell et al., 2016; Rakobowchuk et al., 2017). However, Caldwell et al., reported no change in brachial artery FMD (%) but a decrease in femoral artery FMD (%) after 48 h of eccentric resistance exercise-induced muscle damage (Caldwell et al., 2016). Therefore, the effect of ECC in FMD is still unclear and there might be some confounding factors, such as exercise-induced ESS on artery diameter, affecting endothelial function, which should be considered for better data interpretation.

The purpose of this study was to determine the effect of 30 min of moderate-intensity ECC on endothelial function. We hypothesized that endothelial function would improve after an acute bout of ECC.

## Methods

A convenience sample of 15 college students (9 females, 6 males) was recruited. Inclusion and exclusion criteria defined that the subjects were apparently healthy, normotensive, non-smokers (all tobacco products including e-cigarettes), with no known cardiovascular or cardiac disease, not taking prescribed or over-the-counter medications (excluding birth control) such as acetaminophen or nonsteroidal anti-inflammatories (NSAIDs), took no nutritional supplements containing antioxidants and had no injuries that would affect exercise or ultrasound imaging. All subjects completed two laboratory visits, with 48-h between sessions, in the morning at the same time of the day (±1 h) to avoid any diurnal changes. Females were assessed within 4 days before or after menses to reduce any hormonal influence on vascular reactivity (Adkisson et al., 2010; Mattu et al., 2020). All subjects were asked to abstain from exercise the day before, and they were tested during the morning to avoid circadian variability and following at least 4 h of fasting for the second visist. Additionally, all subjects gave written consent approved by the institutional review board (IRB: 1452826) before scheduling the first visit.

During the first visit, weight and height were measured with a scale and stadiometer (WB-110A Class III, Tanita, Japan; Seca 225, Seca Medical, Germany). Then, subjects performed a cardiopulmonary exercise test (CPET) with a graded exercise test (GXT) protocol on a traditional concentric cycle ergometer (Corival, Lode, Groningen, Netherlands) to determine maximal oxygen consumption (VO_2_max), using a metabolic cart (TrueOne 2400, Parvomedics Inc., Salt Lake City, Utah), peak power output (PPO), and lactate threshold curves (Faude et al., 2009), which were used to determine the exercise intensity for the second test. The seat height was adjusted to allow knee flexion of approximately 5°–15° during cycling. The GXT was a multistage incremental stage starting at 50 W and increasing by 50 W for males and 40 W for females every 2 minutes until exhaustion for approximately 8–12 min in total (Fletcher et al., 2013). The successful measurement of VO_2_max was confirmed by 2 out of 3 criteria: plateau in VO_2_, respiratory exchange ratio >1.1, blood lactate levels from 8–10 mmol/L. Heart rate (HR, Polar H10, Polar Electro, Kempele, Finland), rate of perceived exertion (RPE), circulating blood lactate levels (Lactate Plus, Nova Inc, Waltham, Massachusetts), and Power Output (PO) were collected at the end of each stage. After the CPET on the first visit, a 5-min familiarization stage was implemented on an eccentric upright ergometer (Grucox Medical (Pty) Ltd., South Africa) at 60 rpm and 100 Nm torque.

**FIGURE 1:**
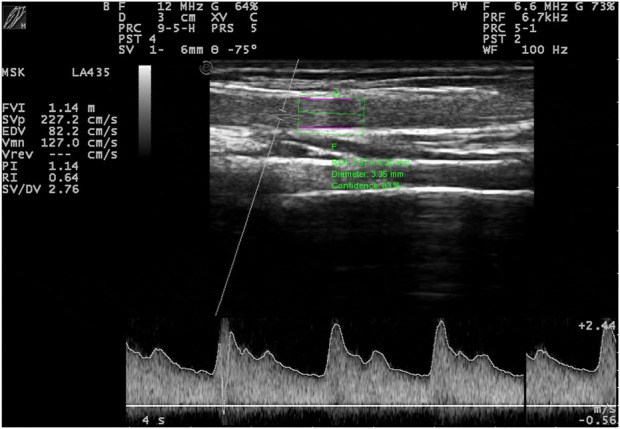
Ultrasound and doppler image of the brachial artery during visit two.

During the second visit, the subjects arrived at the Clinical applied physiology Lab The University of Texas at El Paso between 7 and 11 a.m. then rested in a supine position on an examination table for 10 minutes, the examination room temperature ranged from 20°C–22°C; then, blood pressure was taken three times to establish a resting baseline. FMD measurements (Figure 1) and microhematocrit collection were conducted as previously published by Morales-Acuna (Morales-Acuna et al., 2019). In addition, peak ESS during both FMD assessments was calculated to estimate peak hyperemic stimulus for the FMD using Womersley’s approximation, as previously reported (Gurovich et al., 2021a; Gurovich et al., 2021b; Morales-Acuna et al., 2019). Then, the subjects completed 30-min of moderate intensity, on BLa 2–4 mmol/L, below the lactate threshold, at approximately 60% PPO (Gurovich et al., 2014; Montalvo et al., 2022; Rascon et al., 2020) from the initial GXT, on the eccentric ergometer. In addition, circulating blood lactate levels were recorded every 10 minutes until the end of the exercise bout. Finally, 10 min after completing the 30 min of ECC, FMD measurements were repeated.

### Flow mediated dilation calculations

Pre-FMD% and “traditional post-exercise FMD%” were calculated as previously described in the literature, following the recommendations by Harris et al. (2010). The post-exercise FMD% was adjusted to account for exercise-induced changes to the diameter of the artery post-exercise by using the same baseline diameter from pre-exercise.

Pre-exercise FMD%

$$\frac{PreExercise\;Peak\;Artery\;Diameter-PreExercise\;Baseline\;Artery\;Diameter\;}{Pre\;Exercise\;Baseline\;Artery\;Diameter}\;*\;100$$

Traditional post-exercise FMD%

$$=\frac{PostExercise\;Peak\;Artery\;Diameter-PostExercise\;Baseline\;Artery\;Diameter}{PostExercise\;Baseline\;Artery\;Diameter}\;*100$$

Adjusted post-exercise FMD%

$$=\frac{PostExercise\;Peak\;Artery\;Diamter-PreExercise\;Baseline\;Diameter}{PreExercise\;Baseline\;Artery\;Diameter}\;*\;100$$

Allometric FMDs were calculated as previously described in the literature (Atkinson et al., 2013). Briefly, we determined the Pearson’s correlation r between resting and post-ischemic maximum for pre- and post-exercise. Then, we used the r’s as exponential factors in the FMD calculation below.

$$\text{Pre}‐\text{Exercise}:\frac{PreExercise\;Peak\;Artery\;Diameter}{{{Preexercise\;Baseline\;Artery\;Diameter}^{1}.}^{001}}$$

$$\text{Post}‐\text{Exercise}:\frac{PostExercise\;Peak\;Artery\;Diameter}{{{Postexercise\;Baseline\;Artery\;Diameter}^{0}.}^{89}}$$

### Statistical analysis

Data was extracted from each instrument and compiled into a master data sheet in MS Excel. Data was then exported and analyzed using R statistical programming language (The R Project for Statistical Computing Ver. 4.3.3). Data normality was assessed via visual analysis of a histogram and quantitative means using the Shapiro-Wilk test.

Paired t-tests for normally distributed, or Wilcoxon signed rank-sum test for non-normally distributed data, were utilized to determine the changes in FMD and corresponding variables before and after ECC. When statistically significant, the effect size was determined via Cohen’s d with Hedges’ correction for normally distributed variables and via the Wilcoxon effect size for non-normally distributed data. Statistical significance was set at an alpha level of 0.05.

## Results

### Subject descriptive characteristics

All descriptive data variables except age and weight were normally distributed. Table 1 shows the general characteristics of the sample, represented in the median and interquartile range (IQR). There were no significant differences in age, VO_2_max, HR, lactate, and RPE between sexes (Table 1). Significant differences were found in height and weight between sexes (p < 0.05) (Table 1).

**TABLE 1:** Subject descriptives. Represented in the median and interquartile range.

Subject descriptives			
	Overall	Females	Males
Age (years)	21.9 (3.0)	21.3 (2.0)	22.7 (4.2)
Height (m)	1.6 (0.1)	1.57 (0.05)	1.65 (0.1)
Weight (kg)	62.9 (14.9)	57.1 (8.6)	71.5 (18.8)
VO2 max (mL/kg/min)	32.7 (5.8)	32.3 (5.8)	33 (6.2)
Heart Rate max (bpm)	175 (16)	179 (15)	169 (15)
Lactate max (mmol/L)	6.9 (1.9)	6.7 (1.7)	7.2 (2.2)
RPE max	17 (2)	18 (2)	17 (2)

VO_2_max, Maximal Oxygen uptake; RPE, Rating of Perceived Exertion. *(p < 0.05); between Males and Females.

### FMD variables

**FIGURE 2:**
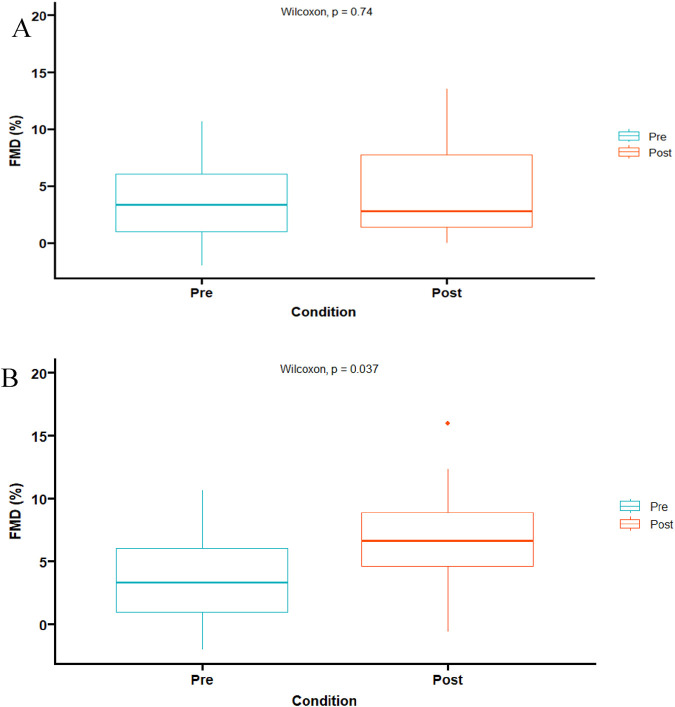
Flow mediated dilation percent (%). **(A)** Using traditional FMD calculations **(B)** Utilizing the same baseline from pre-to post-exercise (p < 0.05).

**FIGURE 3:**
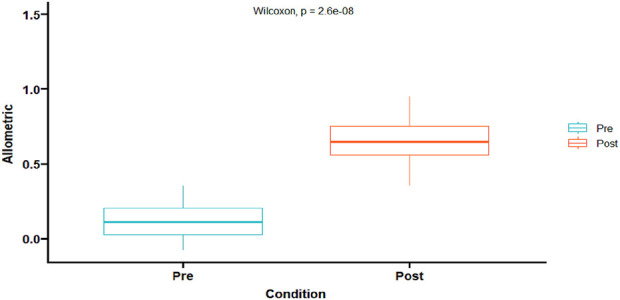
Allometric Scaling differences from pre-to post-exercise. (p < 0.0001).

Data from FMD variables and hyperemic ESS was confirmed to be non-normally distributed. There were no significant differences between pre-and post-exercise in basal and peak diameters (Table 2). In addition, there were no differences in absolute artery diameter difference or FMD between pre- and post-exercise using the traditional method (Table 2; Figure 2A). However, when post-exercise FMD was adjusted to pre-exercise baseline diameter, both post-exercise adjusted absolute artery diameter difference and adjusted FMD were larger than pre-exercise values (p < 0.05) (Table 2; Figure 2B). As well as allometric scaling of FMD resulted in a significant increase post-exercise (Figure 3). Finally, there was no difference in the hyperemic ESS stimulus pre- and post-exercise (Table 2).

**TABLE 2:** Vascular endothelium function parameters.

Vascular endothelium function parameters					
	Pre-exercise (N = 15)	Post-exercise (N = 15)	p	Effect size*	Magnitude
Basal Diameter (mm)	3.60 (0.41)	3.69 (0.48)	0.69	0.076	Small
Peak (mm)	3.73 (0.39)	3.86 (0.46)	0.51	0.125	Small
Absolute difference (mm)	0.13 (0.13)	0.18 (0.14)	0.37	0.167	Small
FMD (%)	3.75 (3.97)	4.60 (4.18)	0.74	0.064	Small
Adjusted Absolute Difference (mm)	0.13 (0.13)*	0.25 (0.15)*	0.026	0.409	Moderate
Adjusted FMD (%)	3.75 (3.97)*	6.97 (4.07)*	0.037	0.382	Moderate
Hyperemic Endothelial Shear Stress	71.6 (17.6)	73.6 (13.2)	0.93	0.026	Small
Allometric	0.12 (0.13)*	0.66 (0.15)*	<0.0001	0.844	Large

FMD, Flow-Mediated Dilation. *(p < 0.05).

## Discussion

The current study aimed to determine the effect of an acute 30-min bout of moderate-intensity ECC on endothelial function. Our findings suggest that vascular reactivity might change 10 min post-ECC. When utilizing the traditional method, no significant changes in endothelial function (FMD%) or absolute difference were observed. However, when adjusting for exercise-induced ESS by adjusting the artery diameter and utilizing allometry, a significant increase in endothelial function (FMD%) and absolute differences were observed.

ECC is a different type of exercise when compared to traditional concentric cycling. When ECC and concentric cycling are performed at the same power output, VO_2_, blood lactate levels, muscle activation, and cardiovascular demand are significantly lower during ECC than concentric cycling (Barreto et al., 2021). Therefore, determining the exercise intensity in ECC is also controversial as metabolic demands and exercise perception do not match (Peñailillo et al., 2014; Peñailillo et al., 2013; Peñailillo et al., 2017). The current study used a combination of metabolic demands (blood lactate levels) and power output to determine exercise intensity for the steady state bout. Moreover, and as previously reported by Montalvo et al., different exercise modalities elicit different blood flow patterns which affect shear rate production (Montalvo et al., 2022), and the increment in antegrade shear rate stimulates the release of NO which can be associated to an improvement on FMD (Dawson et al., 2013). The effect of eccentric exercise on endothelial function remains unclear, with studies reporting a decrease (Haynes et al., 2017; Rakobowchuk et al., 2017; Stacy et al., 2013) or no change (Caldwell et al., 2016). The variability of results reported can be attributed to differences in study design such as sex, exercise intensity, duration, modality, and time-point examination (Rakobowchuk et al., 2017). Finally, baseline diameter and shear rate can also affect the FMD response. Given the challenges of measuring endothelial function via FMD (Thijssen et al., 2011) our group opted for the less studied approach of adjusting post-exercise FMD, using the same pre-exercise baseline diameter for pre- and post-exercise calculations.

This method minimizes the effect of the confounding factor, exercise-induced ESS, of an acute bout of exercise on the artery diameter, similar to Atkinson utilizing an allometric approach (Atkinson et al., 2013). Consequently, when adjusting the artery diameter, a significant increase in both %FMD (Figure 2B) (p = 0.037) and absolute difference (Table 2) (p = 0.026) was observed, as well as allometric scaling (Figure 3) (p = 0.0001). Even though pre- and post-exercise baseline artery diameter and peak post-ischemic artery diameters were not significantly different, there was a 2.5% increase in baseline post-exercise artery diameter, and 3.48% in peak post-exercise artery diameter (Table 2). These small differences, when accounting for exercise-induced ESS, produce significant differences in FMD when adjusting for pre-exercise baseline diameter. Finally, and as shown in Table 2, there were no differences between the hyperemic stimulus between pre- and post-ECC exercise, suggesting that the changes in FMD are mostly associated with the exercise-induced ESS during the ECC bout.

Several studies have reported a decrease in FMD after a bout of ECC exercise (Haynes et al., 2017; Rakobowchuk et al., 2017; Stacy et al., 2013). For instance, Haynes et al. (2017) observed a decrease in endothelial function when investigating short bouts of ECC and concentric cycling matched for workload in males and females with chronic heart failure. Haynes’s study used the traditional FMD assessment, on vascular function, and only when not adjusting for the artery diameter (traditional method) an immediate significant decrease in endothelial function was observed (Haynes et al., 2017). Although Haynes et al. observed a ∼28.5% decrease in endothelial function, a ∼25% increase would have been observed if adjusting for baseline diameter post-exercise was utilized (Supplementary Table S1) (Haynes et al., 2017). Contradictory findings on the endothelial function when utilizing both methods can be attributed to the small differences in baseline (5% increase) and peak (∼2.27% increase) artery diameter during pre- and post-exercise. Additionally, the difference in responses between studies is also mitigated through the difference in exercise prescription and study population. Moreover, the current study and Hayne’s findings contradict when utilizing the traditional but when utilizing the adjusted method, both studies are in agreeance on the effect that ECC can produce enough vasodilation to affect vascular reactivity.

Similar to Haynes et al., Rakobowchuk et al., evaluated the systemic vascular effects of 45 min of continuous ECC at the same aerobic power output as concentric cycling in healthy males and noted a significant decrease in endothelial function 40 min after ECC and an absolute difference in artery diameter (Haynes et al., 2017; Rakobowchuk et al., 2017). Rakobowchuk et al.‘s FMD assessment was collected at three different time points (pre-, 5-min-post, and 40-min-post-exercise) and only at 40-min-post-exercise a significant decrease in endothelial function was observed (Rakobowchuk et al., 2017). Highlighting the complexity of endothelial function, as different recording times may cause differences in its interpretation (Rakobowchuk et al., 2017). Furthermore, Rakobowchuk et al. did not report any significant differences in baseline diameter between pre- and post-exercise but an increase of ∼2% can be observed (Supplementary Table S1), which is similar to the findings of the current study. Interestingly, when adjusting pre-exercise diameter, for absolute diameter change, a decrease of 15% and 100% can be noted 5-min post-exercise and 40-min-post-exercise, respectively. Rakobowchuk et al. (2017) observed a significant decrease (53%) in endothelial function 40-min-post-exercise and a decrease of 15% 5-min-post-exercise when utilizing the traditional method, but when adjusting for baseline diameter, a decrease of 8% 40-min-post-exercise, compared to pre-exercise, and an increase of 30.4% was observed 5-min-post-exercise (Supplementary Table S1). Additionally, when directly comparing the %FMD at the same acquisition time versus adjusted, an increase at both 5-min (54.35%) and 40-min (94.87%) was observed (Supplementary Table S1).

Lastly, the low cardio-respiratory demand used by Rakobowchuk et al., as subjects remained below the first ventilatory threshold, can plausibly explain the decrease in endothelial function. As we have previously shown (Gomez et al., 2024), endothelial shear stress in ECC exercise is intensity dependent for both antegrade and retrograde flows, suggesting that ECC might be a modulator of endothelial homeostasis. Therefore, lower intensities used in previous studies could be insufficient stimulus to enhance endothelial function, while higher intensities, such as the ones used in the current study, could provide an important stimulus to enhance endothelial function (Tinken et al., 2009b). Moreover, remaining below the first ventilatory threshold affect endothelial function as well as the low revolutions per minute (30 rpm) utilized by Rakobowchuk et al. could have influenced the effects of exercise-induced muscle damage (Barnes et al., 2010; Caldwell et al., 2016) on endothelial function. In the current study, we aimed for our subjects to maintain lactate levels under the lactate threshold. Only three subjects had blood lactate concentrations over the lactate threshold. Two out of these three subjects demonstrated both an increase and decrease of endothelial function, regardless of FMD calculation method, where the last one had a decrease in endothelial function in all FMD calculations. Although Rakobowchuck’s subjects remained below the first ventilatory threshold, the current study utilized blood lactate levels as the gold standard for evaluating physiological demands (Gurovich et al., 2018), as lactate levels are closely associated with skeletal muscle metabolism depending on exercise intensity; therefore, the current study demanded higher intensities, which produced higher endothelial shear stress.

Even though the current study only utilized aerobic eccentric exercise, other studies can help to elucidate the differences between exercise modalities. For example, Stacy et al. reported a decrease in FMD % at 1 h, 24 h, 48 h, and 96 h after a bout of eccentric resistance exercise (Stacy et al., 2013). Additionally, Choi et al., reported a decrease in FMD% 45-min after an acute bout of high-intensity eccentric resistance exercise (Choi et al., 2016). Therefore, the decrease in endothelial function noted by Stacy et al. and Choi et al., could be attributed to the exercise-induced muscle damage (EIMD) rather than to any endothelial shear stress modulation. On the one hand, EIMD releases several metabolites that could produce vasodilation (e.g., adenosin, inorganic phosphate, and creatin-kinase) which could be responsible for increasing resting diameters post exercise (Huang and Vita, 2006; Larsen et al., 2015). In all different time points of measurement, the baseline diameter of each time point of recording after pre-exercise, increased by approximately ∼5% (Stacy et al., 2013) (Supplementary Table S1), twice the increase we observed in the current study (Table 2). When utilizing the traditional method, there was a ∼5.5% decrease in all FMD% recordings post-exercise (Stacy et al., 2013), but when utilizing the adjusted method a ∼15% increase in all time points was observed, and most notably an increase of 28% at 24 h post-exercise. Interestingly, Caldwell et al. reported no change of FMD% in the brachial artery 48 h after a bout of knee extensor eccentric exercise (Caldwell et al., 2016). Similarly to Stacy et al. and Choi et al., resistance exercise used for the modulation of endothelial function may not be enough to improve endothelial function. Moreover, the fact that the EIMD was in a different vascular bed than the FMD assessment confirms that local metabolites can modulate vascular function. As Stacy et al., Choi et al., and Caldwell et al. studies used resistance exercise to potentially modulate endothelial function, the low ESS reported by Montalvo et al. suggests that low ESS caused by eccentric resistance exercise on the vascular endothelium might not be a major mitigating factor in the prevention of CVDs (Montalvo et al., 2022). Therefore, the differences in endothelial function reported between studies can be attributed to the differences in volume and intensity utilized in each study. These contradictory findings emphasize the complexity of endothelial function response to an acute bout of eccentric exercise.

In summary, the decrease (Choi et al., 2016; Haynes et al., 2017; Rakobowchuk et al., 2017; Stacy et al., 2013) or no change (Caldwell et al., 2016) of FMD% may not necessarily suggest a reduction in endothelial function but rather an exercise-induced alteration in the brachial artery diameter post-exercise produced by ESS or muscle metabolites (Haynes et al., 2017; Rakobowchuk et al., 2017). Considering all the previous factors, utilizing traditional %FMD calculations to assess the acute eccentric exercise response can be misleading. Considering the evident increase in baseline diameters in all the previous studies, including the present one, with the exception of Choi et al. reporting no change, it is important to acknowledge that post-exercise brachial artery diameter changes are part of broader physiological changes occurring in a cascade, starting with the microcirculation and extending to the macrocirculation. The results of the current study supports using the pre-exercise baseline diameter to avoid the exercise-induced ESS changes on artery diameter, or the muscle metabolites effect on the artery post-exercise, and allometric scaling for post-exercise FMD% calculations.

## Limitations

The present study is not exempt from limitations. Our study was limited by the sample size as well as our sample was only young apparently healthy subjects, as well as not controlling for dietary intake or monitoring mealtimes, only a 4 h fast before the second visit was required. Additionally, the absence of a direct comparison of concentric versus eccentric assessment in the same sample does not allow us for stronger comparisons. Furthermore, and as seen in Figures 2A, B, some participants had a lower-than-expected FMD. However, the majority of them improved after ECC when using both pre-exercise baseline diameter and allometric adjustments.

## Conclusion

The current study showed a significant improvement in endothelial function after 30 min of moderate eccentric cycling when accounting for exercise-induced ESS changes on artery diameter and utilizing allometric scaling. This suggests that for the participants in this study, ECC had a positive effect on endothelial function and may be a beneficial exercise modality for improving CV health. The findings of this study contribute to the growing body of research on the effects of eccentric exercise and provide valuable insights for clinicians and researchers in the field of CV medicine.

## Data Availability

The original contributions presented in the study are included in the article/Supplementary Material, further inquiries can be directed to the corresponding author.
